# Supplementary material for: Uropathogenic Escherichia coli Associated with Risk of Urosepsis—Genetic, Proteomic, and Metabolomic Studies
Source: Int J Mol Sci. 2025 Jun 13;26(12):5681. doi: 10.3390/ijms26125681 (PMC12193174; doi:10.3390/ijms26125681)
Supplement: Supplementary file 1 [file ijms-26-05681-s001.zip › ijms-3670088-supplementary.pdf]

Supplementary material

Uropathogenic Escherichia coli Associated with Risk of Urosepsis—Genetic, Proteomic, and Metabolomic Studies

Beata Krawczyk 1,* , Paweł Wityk 2, Magdalena Burzy ´ nska 1, Tomasz Majchrzak 3
and Michał Jan Markuszewski 2

[1]Department of Biotechnology and Microbiology, Faculty of Chemistry, Gdańsk University of Technology, 80-233 Gdańsk, Poland

[2]Department of Biopharmaceutics and Pharmacodynamics, Medical University of Gdańsk, 80-210 Gdańsk, Poland

[3]Department of Analytical Chemistry, Faculty of Chemistry, Gdańsk University of Technology, 80-233 Gdańsk, Poland;

Table S1. Peptides uniquely identified in urosepsis-derived *E. coli* clinical strains through shotgun proteomics, including associated confidence scores, retention times, and theoretical and observed mass-to-charge (m/z) ratios.

ACCESSIONS	NAMES	BEST CONF (PEPTIDE)	BEST HYPOTH CONF	SEQUENCE	δMASS [DA]	OBS MW [DA]	OBS M/Z [DA]	THEOR MW [DA]	THEOR M/Z [DA]	THEOR Z	ACQ TIME [MIN]	APEX TIME (PEPTIDE) [MIN]	ELUTION PEAK WIDTH (PEPTIDE) [MIN]
SP|P46130|YBHC_ECOLI	ybhC	99	98.49	NLNDTNYNR	-0.0003825	1122.50513	562.2598	1122.50537	562.259949	2	25.86065	25.91	0.33
SP|P0AE88|CPXR_ECOLI	CpxR	97.8	97.79	VLGLELGADDYLPKPFNDRELVAR	0.00406862	2699.43262	675.8654	2699.42822	675.864319	4	51.75218	51.77	0.28
SP|P0AE88|CPXR_ECOLI	CpxR	94.8	89.78	EHLSQEVLGKR	-0.0055461	1294.69385	432.5719	1294.69934	432.57373	3	26.75392	26.75	0.34
SP|P23836|PHOP_ECOLI	PhoP	99	99	VLVVEDNALLR	-0.0112409	1239.70752	620.861	1239.71863	620.866638	2	46.5712	46.62	0.31
SP|P23836|PHOP_ECOLI	PhoP	99	99	SNDVSLPILVLTAR	-0.0064484	1496.84985	749.4322	1496.8562	749.435425	2	54.46332	54.47	0.32
SP|P23836|PHOP_ECOLI	PhoP	96.4	96.39	GQGYLFELR	-0.0029128	1081.55273	541.7836	1081.55566	541.785095	2	47.28715	47.38	0.41
SP|P0AF28|NARL_ECOLI	NarL	94	93.96	IVVFSVSNHEEDVVTALKR	-0.0007042	2141.14746	536.2941	2141.14795	536.294312	4	46.1204	46.14	0.28
SP|P0AF28|NARL_ECOLI	NarL	99	99	ATTERDVNQLTPR	-0.0053686	1499.76392	500.9286	1499.76917	500.930359	3	27.60253	27.62	0.3
SP|P0AF28|NARL_ECOLI	NarL	99	99	LIAQGLPNK	-0.0049473	952.565674	477.2901	952.570557	477.292572	2	31.23493	31.19	0.34
SP|P0AAG3|GLTL_ECOLI	GltL	99	99	VGMVFQHFELFPHLSIIENLTLAQVK	0.00132465	3009.61646	753.4114	3009.61499	753.411011	4	57.0423	56.99	0.46
SP|P0AAG3|GLTL_ECOLI	GltL	99	99	VGLSAHANKFPAQLSGGQQQR	0.0165738	2193.15698	549.2965	2193.14038	549.292358	4	32.01588	32.06	0.34
SP|P05825|FEPA_ECOLI	fepA	99	99	FDHHSIVGNNWSPALNISQGLGDDFTLK	0.00328053	3081.4978	771.3817	3081.49438	771.380859	4	52.24847	52.23	0.28

Table S2. Statistically significant levels of metabolites for the urosepsis and control (UTIs) groups.

NAME	THEORETICAL MASS (M, DA)	OBSERVED MASS (M, DA)	P-VALUE	FDR	AUC	FOLD CHANGE (UROSEPSIS/UTI)
Citric acid	192.027	192.0193	0.0224	0.78652	0.731	0.971
Malic acid	134.0215	134.0119	0.0227	0.75342	0.664	0.793
Ubiquinone-1	250.1205	250.1193	<0.00001	0.98856	0.643	2.963

Table S3. Patients and isolates included in studies.

Patients Group	Genetic studies	Proteomic/metabolomic studies
**Urosepsis group**<br>32 women and 32 men;<br>age of patients: 24-80<br><br>Clinical criteria for urosepsis patients:<br>✓ Body temperature < 36°C or > 38°C;<br>✓ Heart rate > 90 beats per minute; Respiratory rate > 20 breaths per minute or $PaCO_2$ < 32 mmHg;<br>✓ White blood cell (WBC) count < 4 × 10^9/L or > 12 × 10^9/L, with > 10% immature neutrophils.	384 isolates (64 patients) - 3 isolates from blood and 3 isolates from urine per patient for genotyping<br><br>64 isolates from blood –genetic studies<br>(one isolate per one patient)	30 isolates from blood cultivated in arificial urine
**Control group**<br>63 women and 22 men;<br>age of patients: 20-81	85 isolates from urine – genetic studies<br>(one isolate per one patient)	30 isolates from urine cultivated in in artificial urine

Table S4. Genes with sequence primers for PCR assay.

Genes	Function	Sequence primers	Amplicons [bp]	*Ta [C°] In this work	References
**Fimbrial and afimbrial family adhesins**					
fimG/H	fimbriae 1	F: GCAATGTTGGCGTTCGCAAGTGC<br>R: CGTAAATATTCCACACAAACTCC	1001	63	[86]
mrkD	fimbriae 3	F: CCACCAACTATTCCCTCGAA<br>R: ATGGAACCCACATCGACATT	240	60	[88]
papG	fimbriae P	F: GACGGCTGTACTGCAGGGTGTGGCG<br>R: ATATCCTTTCTGCAGGGATGCAATA	328	63	[86]
sfaD/E	fimbriae S	F: CTCCGGAGAACTGGGTGCATCTTAC<br>R: CGGAGGAGTAATTACAAACCTGGCA	410	63	[89,90]
focG	fimbriae F1C	F: CAGCACAGGCAGTGGATACGA<br>R: GAATTCGCCTGCCCATTGCT	360	60	[91]
afa/Dr	Adhesin/fimbriae DR	F: GCTGGGCAGCAAACTGATAACTCT<br>R: CATCAAGCTGTTTGTTCGTCCGCCG	750	60	[89]
tosA (upxA)	RTX protein TosA, afimbrial adhesin (originally annotated as *upxA*)	F: GCACAGCATAACGGGAAAAT<br>R: CCAGCATGTTACCACGAATG	589	60	[92]
**Toxins**					
hlyA	α-hemolysin	F: AACAACGATCCGCACTGTTCTGGCT<br>R: ACCATATAAGCGGTCATTCCCATCA	1177	63	[86, 93]
usp	uropathogenic specific protein	F: TTCTGGGGAACTGACATTCACGG<br>R: CCTCAGGGACATAGGGGGAA	657	63	[86]
cnf1	cytotoxic necrotizing factor 1	F: AAGATGGAGTTTCCTATGCAGGAG<br>R: CATTCAGAGTCCTGCCCTCATTATT	498	63	[93]
**Iron-acquisition systems**					
entB	enterobactin synthesis gene	F: GCGACTACTGCAAACAGCAC<br>R: TTCAGCGACATCAAATGCTC	382	53	[94,95, 96]
fepA	receptor for enterobactin	F: AGCTGACTGACAGCACCATCG<br>R: CGGGATGATCGACAAACGGTCG	554	53	[96]
iroB	salmochelin synthesis gene	F: TATACCGGTCGTGATGCAAA<br>R: ATACTCGGCGGTGTTACGTC	150	54	
iroN	receptor for salmochelin	F: CTTCCTCTACCAGCCTGACG<br>R: GCTCCGAAGTGATCATCCAT	648	54	
iucA		F: ATAAGGGAAATAGCGCAGCA	212	53	

	aerobactin synthesis gene	R: TTACGGCTGAAGCGGATTAC			
iutA	receptor for aerobactin	F: GGCTGGACATCATGGGAACTGG	302	53	
		R: CGTCGGGAACGGGTAGAATCG			
irp-2	yersiniabactin synthesis gene	F: CTGGTGA TGGTGA TGGAAAA	247	54,5	
		R: CCATCGCGATAAATTGTCCT			
fyuA	receptor for yersiniabactin	F: TGATTAACCCCGCGACGGGAA	787	54,5	
		R: CGCAGTAGGCACGATGTTGTA			
chuA	outer membrane hemin receptor	F: ATGGTACCGGACGAACCAAC	288	59	[97]
		R: TGCCGCCAGTACCAAAGACA			
fecA	ferric citrate transport; TonB-dependent outer membrane receptor	F:AGGTTAATATCGCACCGGGATCG	565	60	[67,98]
		R: ATG GCA TCC ATG TTG CCG AGC			
iha	iron-regulated gene homologue adhesion	F: CTGGCGGAGGCTCTGAGATCA	827	60	[95,99]
		R: TCCTTAAGCTCCCGCGGCTGA			
**Autotransporters**					
ag43	autotransporter facilitating biofilm formation	F:GGGTAAAGCTGATAATGTCG	508	60	[100]
		R:GTTGCTGACAGTGAGTGTGC			
aidA-1	AIDA-1 - type V secretion pathway adhesin involved in diffuse adherence	F:TATGCCACCTGGTATGCCGATGAC	600	60	
		R: ACGCCCACATTCCCCCAGAC			
SPATE	serine protease autotransporters of Enterobactericeae	F:GAGGTCAACAACCTGAACAAACGT ATGGG	618	60	
		R:CCGGCACGGGCTGTCACTTTCCAG			
upaG	trimeric autotransporter adhesin (TAA); UpaG bind to ECM proteins	F:CGCGCTCGAGATAATAAGGAATCA ATAATGAACAAAATATT	843	61	[36,54]
		R:CGGCGCTCGAGCATTTGTTAATGG ATGATTACCAC			
upaH	AIDA-I type autotransporter; adhesin involved in diffuse adherence	F:AGTGAAGGGGCAAAAACCTT	328	61	[101]
		R:TGAAACCACCACCATTCTGA			
tosB	serine protease autotransporters (SPATE type)	F: TATCATGTGCTTCAGCCTGGAGGT	150	60	[87]
		R: TCCGTAATATGCCCTGAATCGCCA			
**Protectins and invasins**					
kpsMTII	capsule synthesis	F: GCGCATTTGCTGATACTGTTG	272	60	[91]
		R: CATCCAGACGATAAGCATGAGCA			

ibeA	invasion of brain endothelium A, macrophage survival, inflammatory response	F: AGGCAGGTGTGCGCCGCGTAC	171	60	[102]
		R: TGGTGCTCCGGCAAACCATGC			

*Ta - Temperature annealing used in this work

Table S5. PCR/HaeIII fragment for particular SPATE genes identification. According to Kołowski et al., 2007 [100].

SPATE gene	RFLP/HaeIII [bp]
vat	258, 189, 240, 31
sat	266,189, 97, 66
pic	606, 12
pic-like	384, 168, 66
pssa	294, 256, 68 pz
boa	222, 212, 172, 12
hbp	289, 189, 140

S Figures

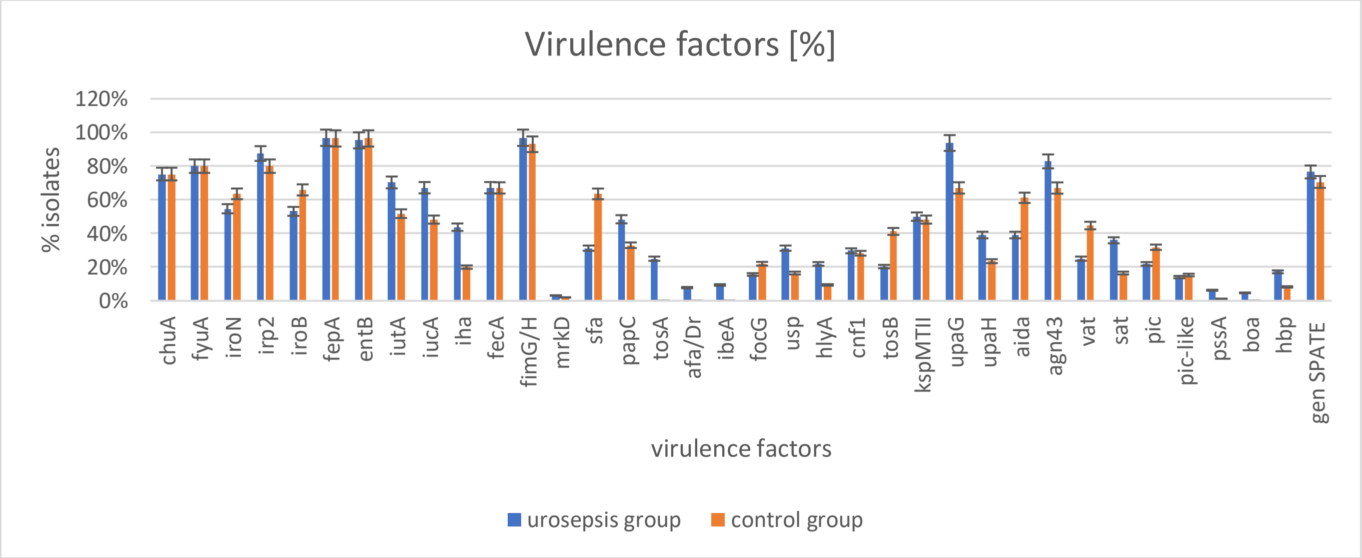

Figure S1. The graphical summary virulence factors for urosepsis and control groups. Error bars - percentage value.

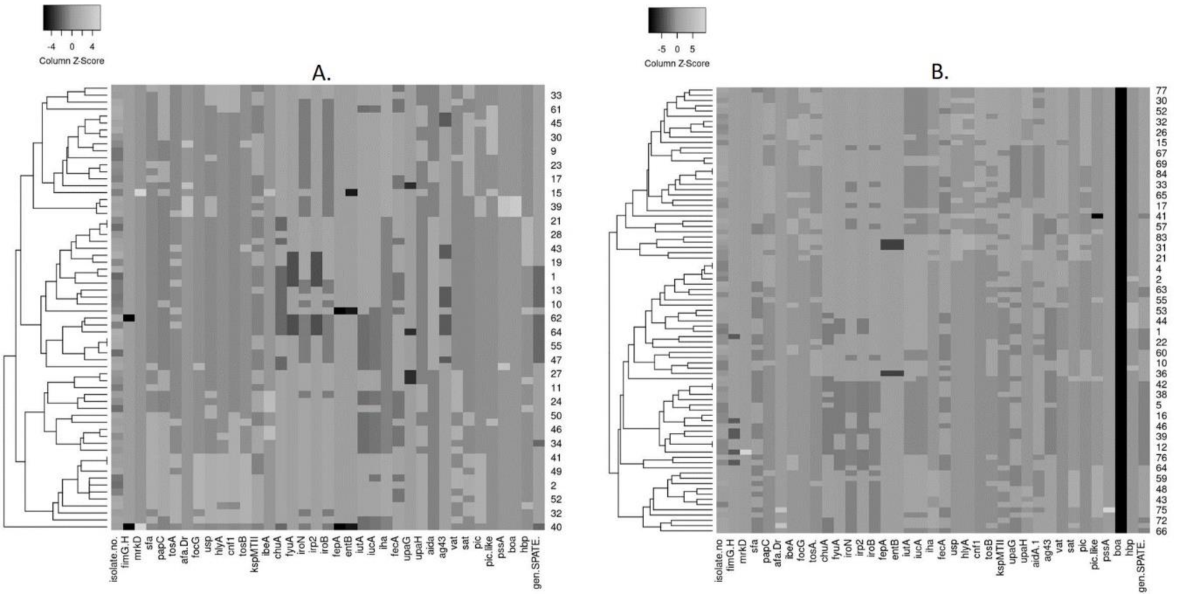

Figure S2. Heatmap of *E. coli* isolates obtained from patients with urosepsis (A, left pane) and from control (urinary tract infection, UTI) cases (B, right pane), based on the distribution of virulence factor (VF) genes. The clustering was performed using the Average Linkage method with Spearman Rank Correlation as the distance metric (generated using Heatmapper: http://heatmapper.ca/expression/). Each row represents a clinical isolate, while each column corresponds to a specific virulence gene. The presence of a gene is indicated by a colored cell, while its absence is shown in darker colors. Hierarchical clustering allowed the identification of isolate groups with similar virulence profiles, suggesting potential pathotype-specific patterns among strains associated with urosepsis. The use of Spearman correlation enabled the analysis to focus on rank-based relationships between gene profiles, which is particularly suitable for binary presence/absence data and non-parametric distributions. The resulting dendrograms (on the left axes) reflect the degree of similarity among isolates and among virulence

factors, revealing potential co-occurrence of gene sets and allowing inference of common virulence mechanisms. A greater diversity was observed among the control (UTI) strains, which clustered into five distinct groups, suggesting higher genetic variability in terms of virulence gene presence. In contrast, urosepsis isolates formed three main clusters, indicating a more homogeneous virulence profile (there seems to be more structured separation among clusters) that may reflect common pathogenic mechanisms associated with bloodstream infections. Inside each cluster, subgroups are included.

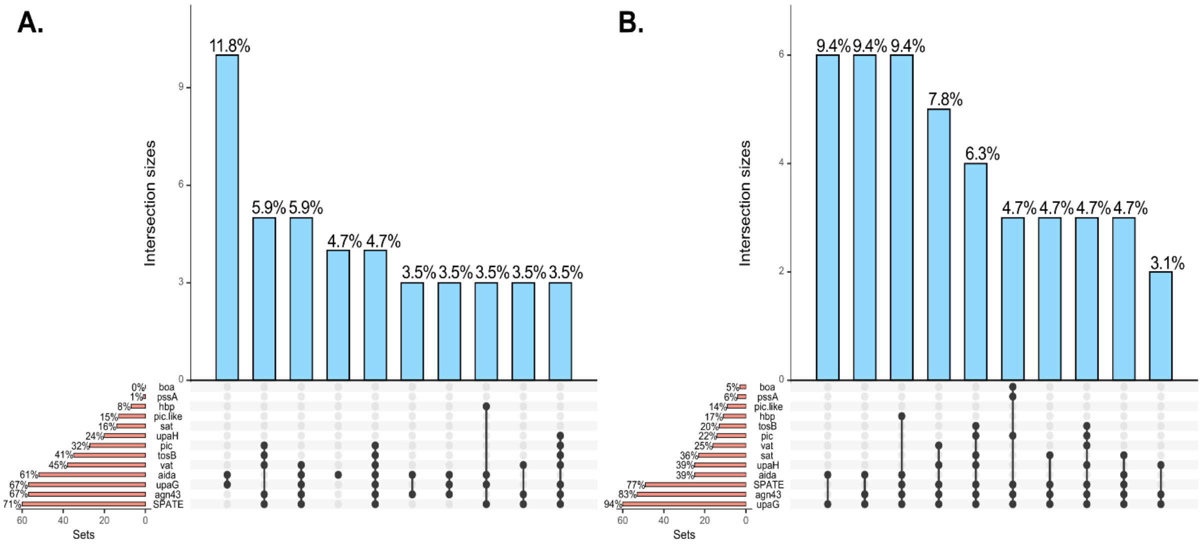

Figure S3. UpSet plots with distribution and coexistence of genes encoding factors involved in receiving and accessing autotransportes. (A) control group, (B) urosepsis group. UpSet plots were generated using R script with UpSetR package (v 1.4.0). Legend: SPATE group (serine protease autotransporters of Enterobacteriaceae); *agn43* - autotransporter facilitating biofilm formation; *aida* - type V secretion pathway adhesin; *upaG* - trimeric autotransporter adhesin (TAA); *tosB, vat, pic, sat, pic-like (U), pssA, boa, hbp* – SPATE group autotransporters. The most frequent combinations in urosepsis isolates were *upaG* + *aidA* and *upaG* + *aidA* + *ag43* (each 9.4%), as well as more complex sets including SPATE and *hbp*. The *boa* gene, exclusive to urosepsis strains, co-occurred with *pssA* in ~5% of cases, suggesting a role in virulence. In contrast, control strains most often carried *upaG* + *aidA* (11.8%), while more complex AT gene combinations were rare.

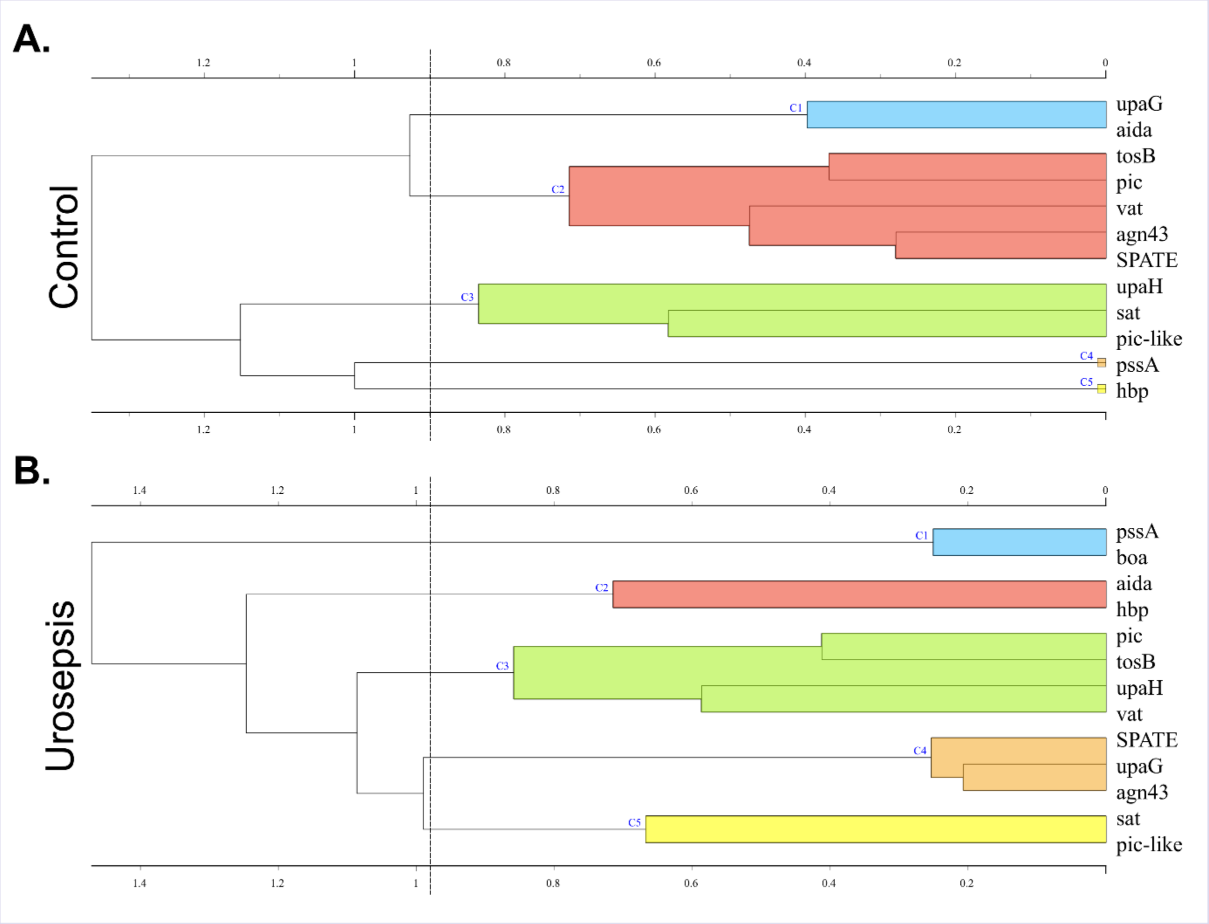

Figure S4. Hierarchical clustering analysis (HCA) of autotransporter (AT) system genes in the control group (A) and the urosepsis group (B). The dendrograms show distinct clustering patterns based on Jaccard distance and Ward linkage. In control strains, two main clusters were identified: one comprising genes associated with lower virulence (e.g., *upaG, aidA, tosB, pic, vat, agn43,* SPATE), and a smaller cluster containing *upaH* and *sat*. In contrast, urosepsis strains exhibited more complex and tighter clusters, including a specific *boa-pssA* cluster, as well as groupings of virulence-related genes such as *aidA*, *hbp*, *pic*, *tosB*, and *vat*, indicating possible co-regulation or synergistic roles in pathogenesis.
